# Supplementary figures and images for: In Vitro Dynamic Model Evaluation of Meropenem Alone and in Combination with Avibactam Against Carbapenemase-Producing Klebsiella pneumoniae
Source: Pharmaceuticals (Basel). 2024 Dec 13;17(12):1683. doi: 10.3390/ph17121683 (PMC11678027; doi:10.3390/ph17121683)

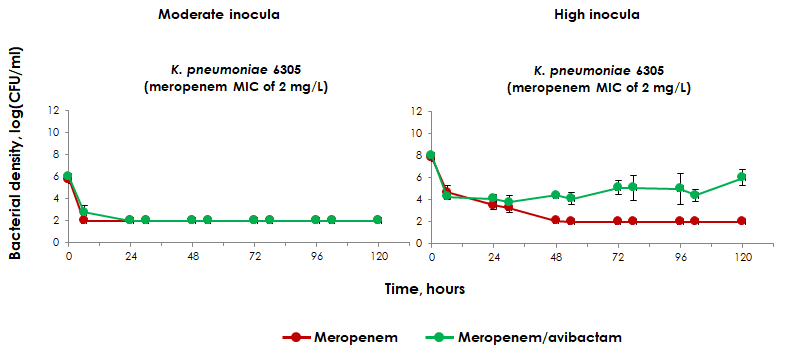

Supplement: Supplementary file 1 [file pharmaceuticals-17-01683-s001.zip › pharmaceuticals-3349405-supplementary.tif]
